# Effect of Controlled Hydrothermal Treatments on Mung Bean Starch Structure and Its Relationship with Digestibility

**DOI:** 10.3390/foods9050664

**Published:** 2020-05-21

**Authors:** Muhammad Awais, Jawad Ashraf, Lili Wang, Liya Liu, Xiaoxue Yang, Li-Tao Tong, Xianrong Zhou, Sumei Zhou

**Affiliations:** 1 Institute of Food Science and Technology, Chinese Academy of Agricultural Sciences/Key Laboratory of Agro-Products Processing, Ministry of Agriculture, Beijing 100193, China; awaismalik74@hotmail.com (M.A.); jawadashraf1@outlook.com (J.A.); wlland2013@163.com (L.W.); liuliya1218@163.com (L.L.); xxyangcass@163.com (X.Y.); tonglitao@caas.cn (L.-T.T.); zhouxianrong@caas.cn (X.Z.); 2 Center for Nutrition and Food Sciences, Queensland Alliance for Agriculture and Food Innovation, The University of Queensland, St. Lucia, Brisbane 4072, QLD, Australia

**Keywords:** mung bean, starch, gelatinization, glycemic index, structure

## Abstract

The changes in structure and digestion properties of mung bean starch due to hydrothermal treatment at various controlled temperatures were investigated. Results showed the increase in onset temperature (T_o_) from 66.33 °C to 76.69 °C and decrease in enthalpies (∆H_g_ and ∆H*_r_*) until the starch was completely gelatinized. The degree of molecular order (DMO) and degree of double helix (DDH) were significantly (*p* < 0.05) reduced from 1.35 to 1.01 and 1.38 to 0.98 respectively. X-ray diffraction (XRD) indicated the consecutive decrease in relative crystallinity (RC) while RVA analysis showed that peak and final viscosities were decreased significantly (*p* < 0.05). However, digestion kinetics indicated that degree of gelatinization increased the access of enzymes. As starch was partially gelatinized it yielded significantly lower glycemic index but no significant (*p* > 0.05) change in starch digestibility was observed after 70 °C. Hence, 70 °C can be considered as the critical hydrothermal treatment temperature in mung bean starch. Pearson’s correlation analysis indicated that controlled hydrothermal treatment had negative effect on the DMO, DDH, RC and the granular damage increased vulnerability of mung bean starch to digestion. These findings gave insight into sequential changes in the structure and digestibility occurring during gelatinization process due to hydrothermal treatment. Controlled gelatinization in mung beans at 70 °C is useful and must be employed to produce the foods with lower starch digestibility.

## 1. Introduction

Mung bean (*Vigna radiata* L.) is a leguminous crop excessively cultivated in South-Asia and it has potential health benefits [1]. It is documented to be rich source of functional components including phytochemicals and proteins. Various bioactivities such as anti-inflammatory, anti-diabetic, anti-tumor and anti-hypertensive have been associated with the mung bean to positively influence the human health [2,3]. Mung bean contains 55–65% starch totaling 630 g·kg^−1^ on dry weight basis which is extracted by employing different processing methods [4]. The size of native starch granules usually varies from 7 µm to 26 µm and it contains much higher amylose contents (up to 45%) as compared to the maximum amylose contents in other cereals, including wheat (35%) and rice (27.2%), respectively. This feature makes the amylolysis of mung bean starch slower and reduces the post prandial glucose responses. Mung bean starch is expensive but an excellent substrate of the food industry and is considered equally important for noodles and vermicelli development [5,6,7,8].

Many structural and functional details of different cultivars of native mung bean starch have been studied by Yao et al. and Gunaratne et al., respectively [9,10]. It tells us about the biodiversity but beans and legumes cannot be consumed directly and need to be cooked before consumption which changes their structure and affects the digestion properties before consumption. Among various botanical origins, starches from beans and pulses are the point of interest because they show lower digestion rates in vitro and in vivo [11]. Therefore, regular consumption of legumes and pulses is proposed to avoid metabolic diseases such as type II diabetes and obesity. The enzymatic hydrolysis of cooked starch largely depends upon the degree of gelatinization [12]. However, some ambiguity still persists about the digestibility mechanisms whether amylolysis of starch increases with gelatinization or the structural orders have implications on digestion rates. The mechanism proposed by Wang et al. [13] says that initial access of amylolytic enzymes is the major determining step in the digestion of starches. This mechanism still needs to be validated in different processing conditions. In a previous study [14], Li et al. investigated the structural features and digestibility in eight different native bean starches but mung bean starch was not included, so keeping in view the prime importance of mung bean starch, it needs to be more thoroughly investigated.

Native starch granules are heated in excess water to convert the highly ordered structure into the disordered structure before consumption, termed as gelatinization. In the process, starch granules undergo series of changes as they absorb and change their volume, viscosity, crystallinity and structural conformations [15,16]. Further multi-scale studies of structure have unveiled that starch consists of an amorphous core which is further surrounded by striated, alternating rings of amorphous and semi-crystalline blocklets. These blocklets contain disordered reducing ends of amylopectin which play significant role in gelatinization and digestion [17,18]. Mung bean starch exhibits solubility and swelling in two different stages. Firstly, in lower temperature range around 50 °C to 70 °C and secondly at higher temperatures above 70 °C to 90 °C. Gelatinized mung bean starch shows gradual increase in solubility and swelling in both phases respectively [19]. This implies that the structural and digestion profiles of mung bean starch in pre-gelatinization and post-gelatinization temperatures are different from each other and still unexplored which largely needs to be investigated. Therefore, further research on gelatinized mung bean starch was performed to understand its structure and digestion properties at the pre and post gelatinization temperatures. To the best of our knowledge, this is the first study of its kind about the structural changes occurring in mung bean starch during the mimicked cooking process (hydrothermal processing) which causes changes in the structure and digestion properties. The structure and digestion properties were studied over both low and high temperature ranges to observe the long-range and short-range starch structures and compared with their relative digestibility. These findings might be helpful for the development of mung bean composites and functional foods with lower starch digestibility.

## 2. Materials and Methods

### 2.1. Materials

The mung bean variety (JLV 8) was purchased from Gucheng Sandou Food Technology Development Co. Ltd., (Heng Shui, Hebei Province, China). Salivary α-amylase (A-3176, Sigma, St. Louis, MO, USA), pepsin (P-6887, Sigma St. Louis, MO, USA), porcine pancreatic enzymes (P-1750, Sigma), amyloglucosidase (E-AMGDF, 3300 U/mL, Megazyme, Bray, Ireland) were used for starch digestion. D-Glucose (k-Gluc, GOPOD format, Megazyme, Bray, Ireland) was used for the glucose measurements. All other reagents used in experiments were of the analytical grade.

### 2.2. Preparation of Starch Dispersions and Heat Treatment

Starch was extracted from mung beans by previous method of extraction [9]. Aqueous dispersions of 5:1 (*v/w*) extracted mung bean starch were prepared at room temperature in polypropylene bags. Starch samples were hydrothermally treated by following the method of Wang et al. [13]. The bags were sealed and given hydrothermal treatment in a water bath at various controlled temperatures i.e., 50 °C, 60 °C, 70 °C, 80 °C, 90 °C and 100 °C for 20 min. Native mung bean starch was taken as a reference sample or control. After heat treatment, the samples were immediately immersed in liquid nitrogen and freeze-dried. Starch samples were subjected to cryogenic milling using ball mill (Retsch 400 MM, Haan, Germany). Milled starch was sieved through 100 µm sieve before further use. Native mung bean starch was taken as control and treated similarly except no heat treatment was applied.

### 2.3. Differential Scanning Colorimetry

Thermal properties of the treated starch samples were analyzed using a differential scanning calorimeter (TA Instruments Inc., New Castle, DE, USA) equipped with thermal analysis data station following the method of Yao et al. [9]. Briefly, 3 mg of starch sample was weighed accurately in an aluminum sample pan and mixed with 10 µL deionized water to hydrate the sample. It was then sealed hermetically and placed overnight at 4 °C for equilibration. The samples were analyzed from 20 to 120 °C with the change in temperature of 10 °C /min. An empty aluminum pan was used as a reference or blank.

Degree of gelatinization (DG) was further calculated by using the previously used formula by [13]. It tells us about the amount of starch crystallites that were melted during heat treatment:

(1) D.G.(%)=(1−ΔHgel./ΔHnat.)×100

where ∆*H_gel_*_._ is the enthalpy changes in gelatinized starch sample and ∆*H_nat_*_._ represents the enthalpy changes in the native starch.

### 2.4. Rapid Viscosity Analyzer (RVA)

The pasting properties were determined using RVA (TecMaster, Perten Instruments, North Ryde BC, NSWAustralia) following the method of Guo et al. [20]. Briefly, 3g of starch sample was weighed precisely and mixed with 25 g deionized water into RVA canister. The standard analysis time was 13 min. After mixing slurry at 960 rpm for 10 s, speed was kept constant at 160 rpm for the rest of experiment. The initial temperature was kept at 50 °C for 1 min and then raised to 95 °C with increment of 6 °C min^−1^ and holding time at 95 °C was 5 min. Then it was cooled down to 50 °C with a decrease of 6 °C min^−1^ and held there for 2 min. The studied parameters included: peak viscosity (PV), trough viscosity (TV), breakdown, final viscosity (FV), and setback.

### 2.5. Starch Morphology

#### 2.5.1. Electron Microscopy

A scanning electron microscope (SEM, S-3400n; Hitachi Ltd., Tokyo, Japan) was used to study morphological changes in the structure of gelatinized mung bean starch granules. Dried starch samples were mounted on SEM stub and pasted on double-sided adhesive tape and sputter-coated with gold in a vacuum evaporator. All the specimens were studied under the accelerating voltage transmission of 10 kV. Images were taken at 500× magnification power (10 µm) and 4500× magnification power (100 µm) for single and grouped starch granules respectively.

#### 2.5.2. Polarized Light Microscopy

A polarization microscope (Motic DMBA 400 China Group Co., Ltd., Guangzhou, China) equipped with a SPOT insight camera was used to analyze the birefringence in starch granules at different degrees of gelatinization in bean starch. The analysis was performed following the previously used method of Li et al. [19]. Mung bean starches with different degrees of gelatinization were mixed with glycerol solution (1:1 *V/V*) and observed under the polarized light microscope at 40× to check the birefringence of the starch.

### 2.6. Wide-Angle X-ray Diffraction

A diffractometer (D8 Advance AXS X-ray powder diffractometer, Bruker, Karlsruhe, Germany) was used to study the crystallinity of dried pre-gelatinized starch samples with moisture content around 10%. All the measurements were performed at 40 kV and 40 mA with Cu-Kα monochromatized radiation with a wavelength (*λ*) of 0.1542 nm. The starch powder was tightly packed in a rectangular glass cell and scanned over the range of 4° to 40° with a function of 2*θ*. The scanning speed during the analysis was 2 °C min^−1^ and scanning step was 0.033°. The relative crystallinity was quantitatively measured by following the method of Yao et al. [9].

### 2.7. Fourier Transform Infrared Spectroscopy

Fourier transform infrared (FTIR) spectra were obtained by using a Fourier transform infrared spectrometer (Nicolet 6700, CA, USA) following the method of [21]. The powdered starch samples were tableted with potassium bromide (KBr) (1/100, mg/mg) ratio and then compressed to form pellet. The pellet was then observed for the absorbance from 40–4000cm^−1^ at a resolution of 4 cm^−1^. Each spectrum was observed for 64 scans at room temperature and the 1:1 ratio of wave numbers 995 to 1022 cm^−1^ and 1045 to 1022 cm^−1^ were calculated to represent the degree of double helix (DDH) and degree of short-range molecular order (DMO) by using the described method of Xu et al. [22].

### 2.8. In Vitro Digestion Kinetics

Extracted native and heat-treated starch samples (50 mg) were dispersed separately in 2 mL of deionized water and incubated with 7.5 mL of sodium acetate buffer (0.2 M, pH 6, 0.49 mM MgCl_2,_ 200 mM CaCl_2_). Then it was placed in shaking incubator at 37 °C for 15 min to attain the temperature. Then a freshly prepared enzyme solution of porcine pancreatic amylase (25 µg) and amyloglucosidase (AMG) was added to the starch suspension. Starch digesta were collected at the regular time intervals (15, 30, 45, 60, 90, 120, 180 min). Enzyme reaction was stopped by the addition of 95% ethanol in the aliquots collected at different time intervals. Undigested starch was removed by centrifugation at 2000× *g* for 5 min at 4 °C. The starch digestibility was calculated by measuring the amount of released glucose by using the kit method (Megazyme Glucose Format Kit). The digestograms were fitted to first-order kinetics as previously described method by Edward et al. [23] and Wang et al. [24] according to Equation (2):

(2) $$C_{t}=C_{\infty }\;\left(1-e^{-kt}\right)$$

where *C_t_* indicates the concentration of digested starch at incubation time *t, C_∞_* is the estimated digestion at infinite endpoint of the reaction and k is the digestion rate coefficient which was measured using a logarithm of slope (LOS) plot analysis using the natural logarithmic form of Equation (2) expressed as Equation (3):

(3) $$\ln\left(\frac{dC}{dt}\right)=-kt+\ln\left(C\infty k\right)$$

where LOS i.e., ln(*dC/dt*) can be plotted against digestion time *t*, which has linear relationship between the slope of *−k* and time t. However, *C_∞_* was calculated by the intercept of the equation and slope *k*.

### 2.9. Statistical Analysis

All the analyses were performed in triplicate and means with standard deviation (S.D.) were calculated. Analysis of variance (ANOVA) was performed by least significance difference (LSD) using SPSS V. 22.0 windows software (SPSS Inc., Chicago, IL, USA). Duncan’s multiple range test was applied and the level of significance was set at *p* < 0.05. Pearson’s correlation analysis was performed to analyze the relationship between structural changes and digestibility of starch.

## 3. Results and Discussion

### 3.1. Thermal Properties

**Figure 1:**
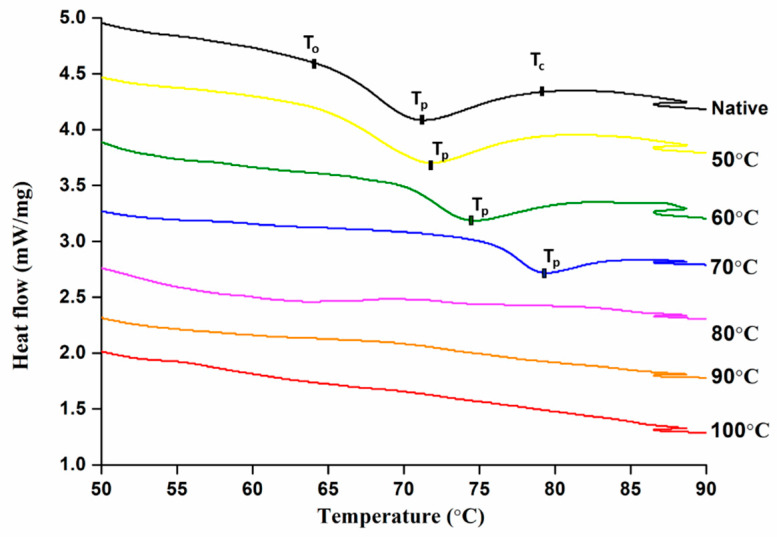
Thermal properties of controlled gelatinized mung bean starch.

Differential scanning colorimetry is an effective tool to measure the endothermic transitions relating to the dissociation of amylopectin and double helices from semi-crystalline to amorphous arrangement. The endothermic values of controlled treated mung bean starch are shown in Table 1. Generally, typical DSC endotherms were obtained up to 70 °C but no endothermic transitions were found above this temperature indicating complete starch gelatinization which is illustrated in Figure 1 below. Firstly, this may be due to the unavailability of crystallites and above 80 °C, the starch was completely gelatinized due to which endotherms were not obtained [9]. Secondly, high temperature could increase the mobility of crystalline and amorphous regions resulting in breakage of inter-linking H-bonds between the adjacent double helices [25].

With the increase in degree of gelatinization (DG), Onset (T_o_), Peak (T_p_), and Conclusion temperatures (T_c_) were increased which can be attributed to the melting of less stable crystallites during the pre-treatment. Hence, higher energy input was required for the dissociation of larger starch crystallites. Similar findings were previously reported about DG and thermal properties by Wang et al. [26], where the thermal properties increased from 56.9 to 64.0 with an increase in DG from 0 to 95.4% at different water to starch ratios respectively. In another study of wheat and potato starch, it was found that only DSC cannot represent the complete gelatinization behavior of starch but represents partial gelatinization due to limited melting and swelling of granules [27].

Enthalpy changes are due to combination of endothermic disruption process of ordered structure and the exothermic process of granular swelling [28]. Contrary to other results, enthalpy of gelatinization (Δ*H_g_*) and enthalpy of retrogradation (Δ*H_r_*) were decreased from 14.07 to 6.77 J·g^−1^ and 10.06 to 6.68 J·g^−1^, respectively, with increasing end temperature of pre-heat treatment. This was attributed to the melting of less stable crystallites initially and later more energy input was required for dissociation of larger crystallites [27].

### 3.2. Pasting Properties

**Figure 2:**
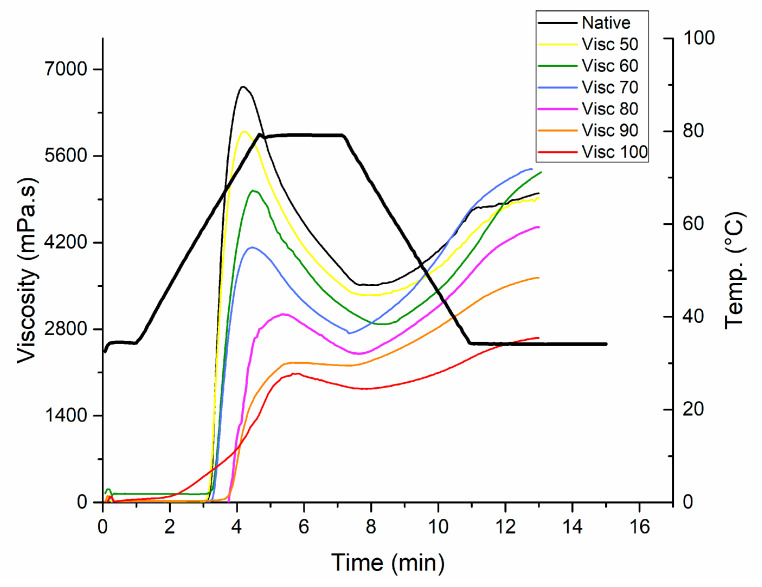
Pasting profiles of controlled gelatinized mung bean starch.

The pasting properties of native and cooked starch samples determined by RVA are shown in Figure 2. The analyzed pasting properties include (peak viscosity, trough viscosity, final viscosity, breakdown, and setback). It can be seen that the heated starch samples showed reduced pasting properties as compared to the native starch. Table 2 shows that peak viscosity reduced significantly (*p* > 0.05) and progressively with increase in thermal pre-treatment temperature from 6613.50 mPa.s to 2015.50 mPa.s from native starch at 100 °C. This can be attributed to maximum damage of starch granules, which was significantly (*p* > 0.05) reduced with the increase of temperature. The rotating paddle was used to measure the resistance of starch during the heating phase which was termed trough viscosity. The trough viscosity was reduced from 3455.50 mPa.s in native starch to 1835.50 mPa.s for maximum gelatinized samples (100 °C) due to loss of inherent capacity of starch granules as surface structure is damaged and water moves inside easily. Hoover et al. (2011) described pasting to be primarily dependent upon swelling characteristics of granules, leaching of granules and chain length distributions (CLD’s) of amylopectin [29].

The point where starch granules are ruptured is termed as breakdown and was calculated from peak and trough viscosity [30]. For native mung bean starch, the pasting temperature is around 70 °C. After pasting, the texture of starch is more strengthened which can be practically utilized for the preparation of thermally stable products. Setback is the limited ability of starch to retrograde. The lower setback values indicate the level of difficulty in retrogradation of starch in native and partially gelatinized samples. However, the samples near the gelatinization temperatures have higher setback values and are prone to the retrogradation phenomenon which can be seen in Table 3. The final viscosity is the viscosity of the cold paste, which is related to the hardness and amylose leached out of the lamellae causing recrystallization [30]. Initially, there was no significant difference in the final viscosity but later the final viscosity decreased with increase in the thermal treatment temperature beyond gelatinization temperature range. It could be due to the damage to crystalline structure and breakage of short amylopectin chains. These findings are consistent with XRD analysis because the pasting profiles were sequentially reduced with the increase in treatment temperature and the samples treated at 90 °C and 100 °C showed collapsed profiles. The results were comparable with a previous study on mung bean starch by Guo et al. [20], in which continuous and repeated dry heat treatments were applied to sweet potato starch and the pasting profiles were reduced with the increase in thermal treatments.

### 3.3. Morphology of Starch

**Figure 3:**
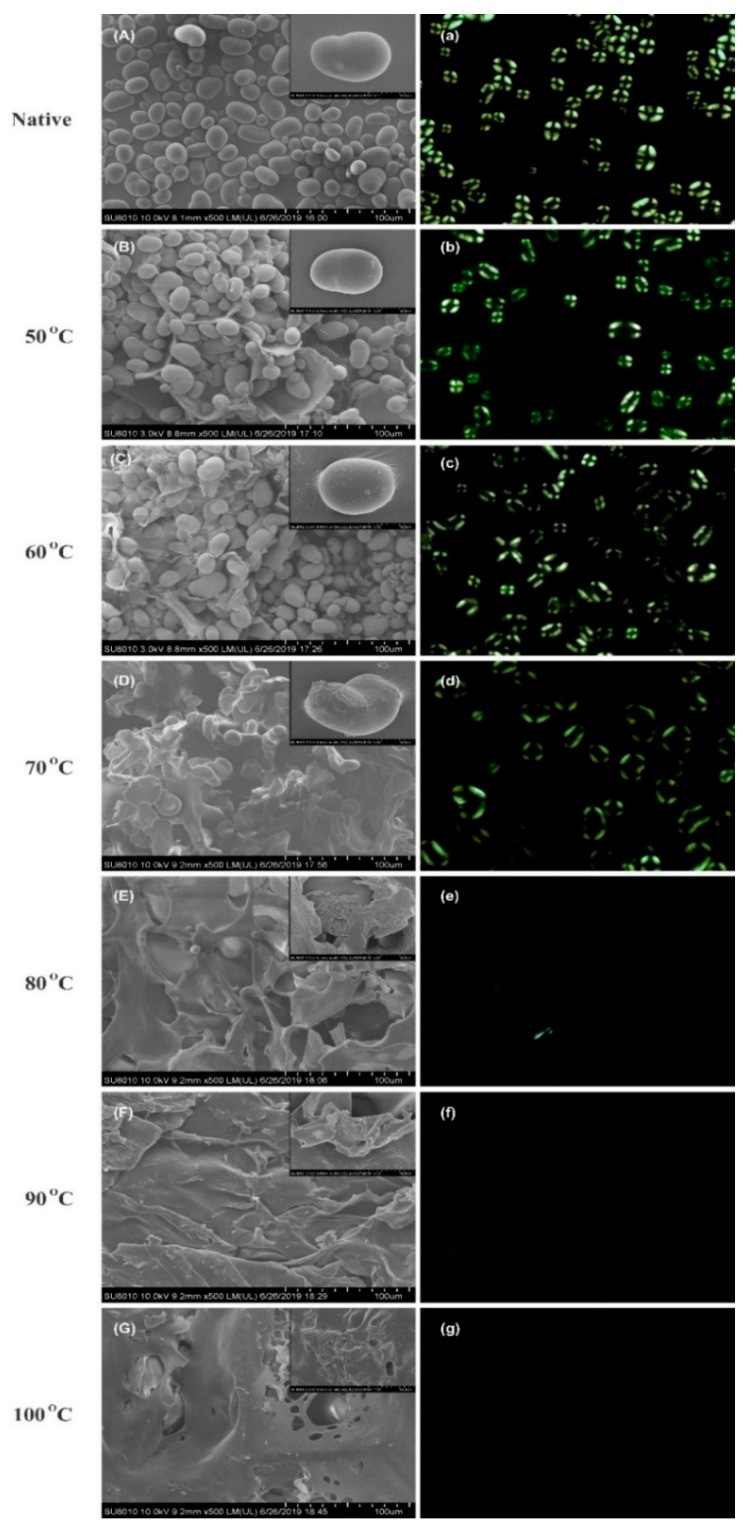
Morphological changes in controlled gelatinized mung bean starch by SEM (**A**–**G**) and Polarized light microscopy (**a**–**g**).

The scanning electron micrographs of cooked starch at different temperatures i.e., 50 °C, 60 °C, 70 °C, 80 °C, 90 °C, and 100 °C are shown in the Figure 3A–G. Mung bean starch demonstrated spherical to elliptical shapes with an average size ranging from 17 to 27 µm and average size was found 22 µm. The measured size was comparable with the range of previously studied mung bean starch i.e., 14 to 27 µm averaging 21 µm [9]. Below gelatinization temperature, no significant differences in the starch morphology were observed. However, the heating at 70 °C caused serious damage to the granular integrity. Furthermore, with each treatment interval, starch granules were disrupted leading to the formation of porous aggregates on surface (Figure 3G). These findings were comparable with the study of Zhang et al. [31], which described gelatinization as the ingress of water molecules into amorphous and crystalline regions exerting disruptive forces in the crystalline region that lead to collapse of the whole structure. Chen et al. studied maize starch gelatinization by hot stage microscopy and indicated that starch granules are swollen during gelatinization process and subsequently break through their cavities and channels [32]. Later on, it was revealed that gelatinization starts at the botanical center (hilum) which is less organized structure and then spreads outwards to the periphery [33]. Figure 3a–g shows the birefringence patterns of gelatinized mung bean starch granules heat-treated at different temperatures. Birefringence can be defined as the average radial orientation of the double-helical structures. A typical birefringence exists in native starch granule in which complete and clear Maltese crosses can be observed [19]. The 50 °C is said to be the onset temperature because with the progression of gelatinization, the birefringence started to become blurred. Eventually, above the gelatinization temperature, 80 °C can be termed as endpoint temperature because the birefringence was completely lost. The loss of these maltese crosses is consistent with the loss of granular structure in micrographs of electron microscopy. In a previous study by Li et al. [19], ultra-high pressure was applied for the gelatinization of mung bean starch and the birefringence was completely lost at 600 mPa pressure. Contrary to this, heat-moisture treatments in potato starch showed complete loss of birefringence at 110 °C at 21 and 24% moisture level [34].

### 3.4. X-ray Diffraction

**Figure 4:**
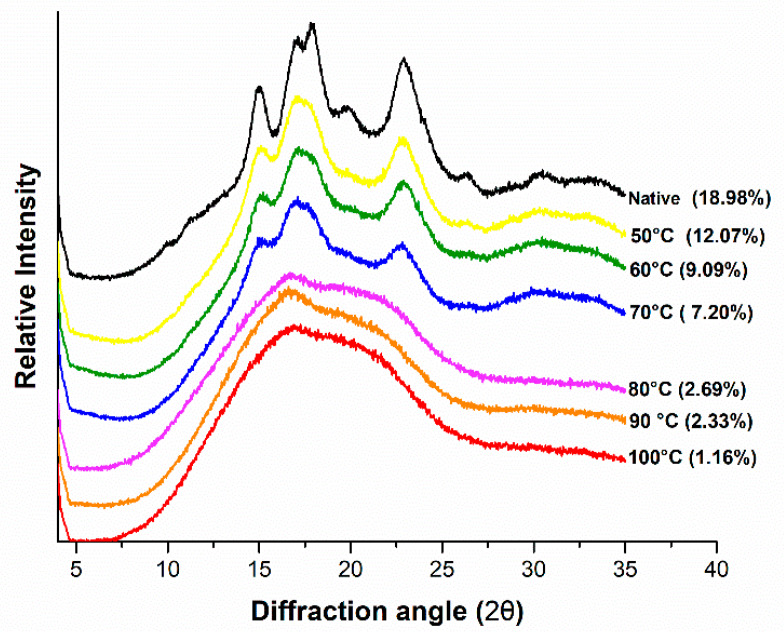
XRD patterns of controlled gelatinized mung bean starch.

X-ray diffraction profiles of gelatinized mung bean starch with relative crystallinity (%) at various temperatures are shown in Figure 4. Mung bean starch showed C-type crystallinity pattern characterized by five reflection intensities of 2θ around 15.00°, 17.00°, 17.83°, 19.83° and 22.91°. Similar reflection peaks were reported by Hoover et al. [35], in native mung bean starch sample. The minor peak obtained at 19.83° indicated the amylose-lipid complex in mung starch which was highest in native sample but reduced greatly with increasing the temperature [36]. This was possibly due to the leaching of amylose from the lattice with the thermal treatment. The relative crystallinity calculated on the basis of obtained diffraction patterns is shown in Figure 4. Native mung bean starch exhibited highest relative crystallinity (18.98%). It was within the range of 17.23% to 19.41% previously recorded by Yao et al. [9]. Hydrothermal treatment reduced the relative crystallinity to 12.07%, 9.09%, 7.20%, 2.69%, 2.33% and 1.16% due to gelatinization at 50, 60, 70, 80, 90 and 100 °C, respectively. The relative crystallinity of native mung bean starch was recorded much lower as compared to eight other bean starches from lowest in great split bean (27.9%) to highest in Pinto bean (35.1%) [14]. This difference was attributed to crystal size, amounts of crystalline regions, orientation of double helices and extent of interactions between double helices [37].

### 3.5. Short-Range Molecular Order by Fourier Transform Infrared Spectroscopy

**Figure 5:**
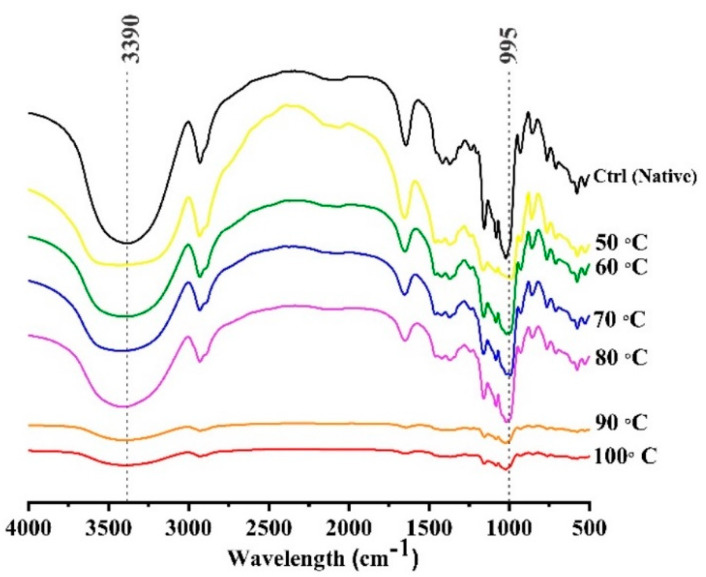
FTIR spectra of controlled gelatinized mung bean starch.

The infrared spectra have been displayed in Figure 5. The infrared spectral ratios at 1047/1022 cm^−1^ and 995/1022 cm^−1^ can be related to the degree of short-range molecular order and degree of long-range double helix, respectively [38]. The short-ordered structure can be described as a measure of the ratio between amorphous and crystalline structure of starch. It was observed that cooking treatment resulted in the significant disruption of crystalline and amorphous structures in mung bean starch. However, the absorption peaks from 3000 to 3600 cm^−1^ shown as 3390 cm^−1^ in Figure 4, are associated with the vibrational stretching of hydroxyl groups. These peaks were decreased relating to the inter or intra hydrogen bonding within or amongst the starch granules. In 90 and 100 °C samples, the hydrogen bonds were partially disrupted which led to the decrease of absorption peak at 3390 cm^−1^. Table 2 shows that calculated values for degree of molecular order (DMO) and degree of double helix (DDH) were significantly reduced from 1.35 and 1.38 to 1.01 and 0.98 respectively. The disruption of DMO until 70 °C was much slower as compared to the pre-gelatinized samples but the short-range ordered structure was still present at highest temperature of gelatinization treatment (100 °C). Generally, the FTIR absorption peaks were in the decreasing trend until 80 °C but they were collapsed at 90 °C and no further noticeable difference was observed at 100 °C. It is previously reported in wheat starch that crystalline structure was melted earlier as compared to molecular orders [33]. It can be observed in findings of this study that at 80 °C, the crystalline peaks were greatly reduced but the amorphous region still has pronounced peaks at 995 cm^−1^. The short-range ordered structure was still present at the maximum heat treatment temperature (100 °C). The results were comparable with Xu et al. [22], where DMO and the O-H stretching were decreased significantly (*p* > 0.05) between the native and autoclaved pea and navy bean samples, which was attributed to the uncoiling of the double-helical structures of starch granules.

### 3.6. In Vitro Digestion Kinetics

**Figure 6:**
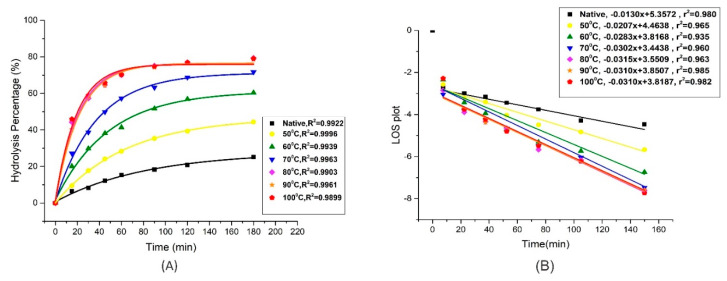
Digestograms of controlled gelatinized mung bean starch showing (**A**) Non-linear curve fitting and (**B**) LOS plots.

In vitro digestion kinetics of gelatinized mung starch to different degree of gelatinization are given in Table 3. The progression curve graph in Figure 6A indicates that the digestion was rapid in the first hour of the experiment but afterwards it was delayed when the concentration of starch was exhausted. As it is already known that the major digestion limiting factors include the origin of starch and the concentration of the enzymes used in the digestion process [39]. The native starch sample followed a more or less linear fit but the gelatinized samples showed curved progression fitting. The k values are the indicators of digestion rate coefficients which were lowest in native starch and increased with increase in degree of gelatinization (DG) from 0 to 100% respectively. The lowest k values of native starch indicated that it was digested slowly and with the increase in heat treatment, the starch was digested at a faster pace. The digestibility of native mung bean starch was found to be within the range of 19.6 to 28.6 mg/g equivalents of maltose among established and newly developed mung bean cultivars [40]. The k value was obtained by fitting the enzyme catalytic data to the LOS plot as previously done by Butterworth et al. [41] is shown in Figure 6B. The k value was increased from 0.015/min to 0.041/min when DG increased from 0% to 100%. Similarly, in the study of Wang et al. [26], *k* values were increased 2.5-fold between native and the gelatinized sample. The digestion extent (*C_∞_)* at the endpoint of reaction varied significantly (*p* < 0.05) from native (22.69 ± 0.50%) to 78.99 ± 0.10% for samples gelatinized at temperatures up to 80 °C. However, no significant difference was observed when the starch was completely gelatinized in heat-treated samples at 80 °C, 90 °C, and 100 °C. Similar results were reported by Wang et al. [13], where the susceptibility of potato and lotus seed starch breakdown by enzymatic hydrolysis was increased due to the increase in gelatinization. Similarly, in wheat starches, the cooked samples were digested rapidly as compared to native and may result in higher glycemic and insulinemic responses in body [36]. The accountable reason was said to be the damaged starch granules and the internal crack of hilum. However, Li et al. listed disruption of double-helical structure and crystallinity to be the determinants in the digestion of starches which is in accordance with our findings from FTIR and XRD as they also indicated the disruption in double helical structure and crystallinity [14]. The relation between starch structure and digestion is complex which depends on various factors such as amylose content, orientation of amylopectin, degree of crystallization and the particle size [42]. Keeping in view these factors, processing of foods is now focused on maintaining the intact cells encapsulated within cell walls using different processing technologies. This hinders the gelatinization process and retains the ordered structures of starches resulting in lower enzyme susceptibility [43].

### 3.7. Pearson’s Correlation Analysis

The values of Pearson’s correlation analysis are shown in Table 4. It indicated that Onset temperature (T_o_) was highly positively correlated with all other DSC parameters (T_p_, T_c_, ∆*H_g_* and ∆*H_r_*) but degree of gelatinization (DG) was found to be negatively correlated (*p* < 0.01). This indicates that the more starch is pre-treated or gelatinized, the lesser will be its thermal viability. The digestion extent (*C_∞_)* and the value of k were found strongly correlated (0.898 **) and (0.997 **) with the degree of gelatinization (DG) respectively. This suggests that the pre-gelatinized starch tend to digest more easily due to damage of granular structure and easier initial binding of amylases to the starch.

The structural parameters such as degree of molecular order (DMO), degree of double helix (DDH) and relative crystallinity were found to be strongly negatively correlated with the digestion extent (*C_∞_).* The controlled thermal treatment of starch lead to the decrease in the structural orders and increase in the digestion extent of the starch. These findings were found contrary to the results of Li et al. (2017) [14], where no correlation was observed between the structural parameters of native bean starches and their digestibility. A strong positive correlation was observed in the viscosity (P.V. and T.V.) and the enthalpies of gelatinization and retrogradation (∆*H_g_* and ∆*H_r_*_)_.

## 4. Conclusions

Controlled gelatinization of Mung bean starch was carried out at different temperatures and the transitional changes in the digestibility and structure were observed. The structure-digestion relationship was observed by Differential scanning colorimetry (DSC), Scanning electron microscopy and short-range molecular orders. The results indicated that 70 °C was the ideal gelatinization temperature for lower glycemic index but at further gelatinization, the structure was disrupted and in-vitro digestibility was maximum. Mung bean products especially noodles can be cooked at 70 °C for slightly longer time to reduce the glycemic index. Moreover, mung bean composites can be prepared with other key cereals to reduce the glycemic index and insulinemic responses. Correlation analysis indicated that controlled gelatinization treatment had negative effect on the amorphous and crystalline orders of starch structure and the granular damage increased the vulnerability of mung bean starch to digestion. It was found that controlled gelatinization was useful to control the digestibility of starch.

Various other methods of thermal treatments such as microwave and roasting can be used to control the degree of gelatinization in the future to get the lower starch damage and lower starch digestibility.

## Figures and Tables

**Table 1:** Thermal properties of controlled gelatinized mung bean starch.

Sample	Onset	Peak T.	E. of Gel.	Concl. T.	E. of Ret.	DG
	T_o_	T_p_	ΔH_g_	T_c_	ΔH_r_ = Tc − T_o_	(%)
Native	66.33 ± 0.46 ^c^	71.71 ± 0.36 ^c^	14.07 ± 1.46 ^a^	76.39 ± 0.04 ^c^	10.15 ± 0.41 ^a^	0
50 °C	66.48 ± 0.00 ^c^	71.62 ± 0.43 ^c^	14.40 ± 1.81 ^a^	76.49 ± 0.36 ^c^	10.06 ± 0.36 ^a^	2.5
60 °C	70.01 ± 1.58 ^b^	73.87 ± 1.87 ^b^	11.44 ± 2.39 ^a^	78.61 ± 2.25 ^b^	8.60 ± 0.60 ^b^	20.55
70 °C	76.69 ± 0.03 ^a^	79.84 ± 0.04 ^a^	6.77 ± 2.05 ^b^	83.38 ± 0.62 ^a^	6.68 ± 0.65 ^c^	52.98
80 °C	N.D.	N.D.	N.D.	N.D.	N.D.	100
90 °C	N.D.	N.D.	N.D.	N.D.	N.D.	100
100 °C	N.D.	N.D.	N.D.	N.D.	N.D.	100

where T_o_, Tp, and Tc, indicate the Onset, Pasting and Conclusion temperature but Enthalpy of gelatinization and Enthalpy of retrogradation are expressed as Δ*H_g_* and Δ*H_r_* respectively. Mean values ± S.D. in the same column followed by different superscript letters are significantly different (*p* < 0.05).

**Table 2:** Pasting profiles of controlled gelatinized mung bean starch.

Sample	P. V.	T. V.	F. V.	Bd.	Sb.
	(mPa.s)	(mPa.s)	(mPa.s)	(mPa.s)	(mPa.s)
Native	6613.50 ± 121.62 ^a^	3455.50 ± 82.73 ^a^	4784.50 ± 286.37 ^c^	3157.50 ± 38.89 ^a^	1329 ± 203.64 ^d^
50 °C	5846 ± 192.33 ^b^	3307.50 ± 96.87 ^ab^	4866.50 ± 71.41 ^bc^	2538.50 ± 95.45 ^b^	1559 ± 25.45 ^d^
60 °C	4906 ± 38.18 ^c^	2833.50 ± 109.60 ^c^	5105 ± 103.23 ^ab^	2072.50 ± 71.41 ^c^	2271.50 ± 212.83 ^b^
70 °C	4067 ± 57.98 ^d^	2781.50 ± 65.76 ^c^	5390 ± 32.52 ^a^	1285.50 ± 123.74 ^d^	2608.50 ± 33.23 ^a^
80 °C	3027.50 ± 35.50 ^e^	2395.50 ± 26.50 ^cd^	4435 ± 21.00 ^c^	631.5 ± 9.5 ^e^	2039 ± 5.00 ^c^
90 °C	2244 ± 18.38 ^f^	2214 ± 25.45 ^d^	3612.50 ± 24.74 ^d^	30 ± 7.07 ^f^	1398.50 ± 0.70 ^d^
100 °C	2015.50 ± 75.66 ^f^	1835.50 ± 9.19 ^e^	2650.50 ± 10.60 ^e^	180 ± 66.46 ^f^	815 ± 1.41 ^e^

Peak viscosity, trough viscosity, final viscosity, breakdown, and setback are abbreviated as P.V., T.V., F.V., Bd., and Sb., respectively. Mean values ± S.D. in the same column followed by different superscript letters are significantly different (*p* < 0.05).

**Table 3:** Digestion and FTIR properties of controlled gelatinized mung bean starch.

Sample	C_∞_	K	DMO	DDH
	(%)	(min^−1^)	(1047/1022 cm^−1^)	(995/1022 cm^−1^)
Native	22.69 ± 0.50 ^e^	0.015 ± 0.000 ^cd^	1.35 ± 0.0002 ^a^	1.38 ± 0.001 ^a^
50 °C	43.34 ± 1.02 ^d^	0.018 ± 0.000 ^c^	1.17 ± 0.0001 ^b^	1.25 ± 0.0002 ^b^
60 °C	60.09 ± 0.30 ^c^	0.022 ± 0.000 ^bc^	1.06 ± 0.0002 ^c^	1.07 ± 0.0000 ^c^
70 °C	71.70 ± 0.01 ^b^	0.030 ± 0.001 ^b^	1.04 ± 0.001 ^d^	1.06 ± 0.0035 ^d^
80 °C	79.16 ± 0.17 ^a^	0.040 ± 0.004 ^a^	1.04 ± 0.002 ^d^	1.00 ± 0.0004 ^e^
90 °C	78.70 ± 0.39 ^a^	0.041 ± 0.001 ^a^	1.03 ± 0.0006 ^e^	0.9918 ± 0.0004 ^f^
100 °C	78.99 ± 0.10 ^a^	0.041 ±0.000 ^a^	1.01 ± 0.0008 ^f^	0.98 ± 0.0003 ^f^

Degree of molecular order, degree of double helix, the end-point concentration of digested starch and k-constant values are expressed as DMO, DDH, C_∞_ and k, respectively. Mean values ± S.D. in the same column followed by different superscript letters are significantly different (*p* < 0.05).

**Table 4:** Correlation analysis between structural changes and digestibility of starch.

	T_o_	T_p_	ΔH_g_	T_c_	∆H_r_	DG	C_∞_	K	DMO	DDH	P. V.	T. V.	F. V.	Bd.	Sb.
**T_p_**	1.000 **	1													
**ΔH_g_**	0.891 **	0.901 **	1												
**T_c_**	0.999 **	1.000 **	0.907 **	1											
**∆H_r_**	0.946 **	0.954 **	0.990 **	0.958 **	1										
**DG**	−0.891 **	−0.901 **	−1.000 **	−0.907 **	−0.990 **	1									
**C_∞_**	−0.665	−0.682	−0.890 **	−0.69	−0.849 *	0.898 **	1								
**K**	−0.874 *	−0.885 **	−0.995 **	−0.891 **	−0.982 **	0.997 **	0.923 **	1							
**DMO**	0.521	0.538	0.75	0.545	0.708	−0.762 *	−0.963 **	−0.803 *	1						
**DDH**	0.645	0.661	0.859 *	0.668	0.821 *	−0.865 *	−0.988 **	−0.891 **	0.975 **	1					
**P.V.**	0.840 *	0.852 *	0.968 **	0.857 *	0.955 **	−0.972 **	−0.938 **	−0.984 **	0.853 *	0.923 **	1				
**T.V.**	0.832 *	0.843 *	0.935 **	0.847 *	0.929 **	−0.937 **	−0.898 **	−0.947 **	0.831 *	0.905 **	0.980 **	1			
**F.V.**	0.834 *	0.830 *	0.688	0.829 *	0.749	−0.687	−0.461	−0.681	0.375	0.465	0.722	0.785 *	1		
**Bd.**	0.832 *	0.844 *	0.971 **	0.850 *	0.954 **	0.975 **	−0.943 **	−0.988 **	0.851 *	0.919 **	0.995 **	0.957 **	0.682	1	
**Sb.**	0.509	0.494	0.186	0.487	0.286	−0.183	0.132	−0.164	−0.202	−0.131	0.196	0.275	0.812 *	0.155	1
**R.C.**	0.774 *	0.787 *	0.919 **	0.792 *	0.903 **	0.928 **	−0.979 **	−0.951 **	0.938 **	0.968 **	0.969 **	0.944 **	0.607	0.968 **	0.053

** Correlation is significant at the 0.01 level (*p* < 0.01); * Correlation is significant at the 0.05 level (*p* < 0.05) where T_o_, T_p_, Δ*H_g_*_,_ T_c,_ ∆*H_r_*, DG, C_∞,_ K, DMO, DDH, P.V., T.V., F.V., Bd., Sb., and R.C. represent Onset temperature, Peak temperature, Enthalpy of gelatinization, Conclusion temperature, Enthalpy of retrogradation, Degree of gelatinization, Endpoint concentration of digested starch, K-constant, Degree of molecular order, Degree of double helix, Peak viscosity, Trough viscosity, Final viscosity, Breakdown, Setback and relative crystallinity respectively.
